# Estimation for Runway Friction Coefficient Based on Multi-Sensor Information Fusion and Model Correlation

**DOI:** 10.3390/s20143886

**Published:** 2020-07-13

**Authors:** Yadong Niu, Sixiang Zhang, Guangjun Tian, Huabo Zhu, Wei Zhou

**Affiliations:** School of Mechanical Engineering, Hebei University of Technology, Tianjin 300130, China; 201811201020@stu.hebut.edu.cn (Y.N.); zhangsx@hebut.edu.cn (S.Z.); 201821203018@stu.hebut.edu.cn (G.T.); 201821203019@stu.hebut.edu.cn (H.Z.)

**Keywords:** tire–runway friction, multi-sensor information fusion, sensor system, neural network, ground friction coefficient, aircraft braking friction coefficient, correlation model, mobile weather–runway–tire system

## Abstract

Friction is a crucial factor affecting air accident occurrence on landing or taking off. Tire–runway friction directly contributes to aircraft stability on land. Therefore, an accurate friction estimation is a rising issue for all stakeholders. This paper summarizes the existing measurement methods, and a multi-sensor information fusion scheme is proposed to estimate the friction coefficient between the tire and the runway. Acoustic sensors, optical sensors, tread sensors, and other physical sensors form a sensor system that is used to measure friction-related parameters and fuse them through a neural network. So far, many attempts have been made to link the ground friction coefficient with the aircraft braking friction coefficient. The models that have been developed include the International Runway Friction Index (IRFI), Canada Runway Friction Index (CRFI), and other fitting models. Additionally, this paper attempts to correlate the output of the neural network (estimated friction coefficient) with the correlation model to predict the friction coefficient between the tire and the runway when the aircraft brakes. The sensor system proposed in this paper can be regarded as a mobile weather–runway–tire system, which can estimate the friction coefficient by integrating the runway surface conditions and the tire conditions, and fully consider their common effects. The role of the correlation model is to convert the ground friction coefficient to the grade of the aircraft braking friction coefficient and the information is finally reported to the pilots so that they can make better decisions.

## 1. Introduction

The aircraft taxiing on runways relies on the friction generated by the tires and the runway surface, which enables the aircraft to safely accelerate, turn, taxi, and eventually stop the aircraft from moving. Recent years have seen a frequency of accidents involving an aircraft rushing out of the runway on landing or taking off. In the past two years, there have been many accidents. On May 3, 2019, a plane, carrying 140 people in the United States, ran out of the runway and slipped into a river. On June 30 and July 1, 2019, there were two consecutive days of aircrafts rushing out of the runway in India. On February 5, 2020, a Turkish aircraft crashed out of the runway during a hard landing and injured 179 people. The Civil Aviation Administration of China found out that the accident rates in severe weather conditions were much higher than that in ordinary, mainly because the friction had not been correctly estimated [1]. Due to human casualties and economic losses, many countries have devoted numerous researches to develop measuring methods or tools for accurately measuring runway friction in all weather conditions [2]. Therefore, an accurate friction estimation is a rising issue for all stakeholders.

### 1.1. Problem Statement

Up to now, there have not been many studies on the airport runway friction measurement, and airports mainly use a surface friction tester (SFT) to measure the runway friction coefficient. This test takes into account the combined effect of the tire and the wet runway surface, but this expensive equipment and high failure rate increase the cost of an airport, meanwhile, the measurement accuracy has a great relationship with the operator’s operation. Some methods are proposed for the friction research between tire and road surface. However, some of them estimate the friction coefficient based on the tire characteristics (such as tire pressure, load, and deformation), while others estimate the friction coefficient based on the road surface characteristics (such as surface texture, temperature, and contaminant type). They all seem to ignore each other’s contribution to friction.

### 1.2. Objectives of Research

The main objective of this paper is to propose a scheme that can be used to estimate the airport runway friction coefficient. The core of the scheme is to build a sensor system that can measure both runway surface information and tire information. In this paper, the joint action of the two is comprehensively considered to improve the estimation accuracy of the friction coefficient. Further, the correlation method is proposed to predict the aircraft braking performance and report it to the pilots, which helps them make good decisions when landing.

### 1.3. Summary of Contributions

The following contributions are reported in this research. A summary of each method for measuring the friction coefficient is given first. Secondly, according to the merits of the existing friction measurements, an estimation scheme based on multi-sensor information fusion is proposed. Sensors such as optics and treads are used as an integrated sensor system, which is installed on a test car to make it become a mobile weather–runway–tire system. Due to its mobility, the sensor system can measure information about the friction characteristics at any position of the entire runway. The sensor system is integrated by different types of sensors, so the neural network is selected as the fusion algorithm. Finally, this paper attempts to correlate the relationship between the ground friction measurement and the aircraft braking friction coefficient. Existing correlation models include International Runway Friction Index (IRFI), Canada Runway Friction Index (CRFI), NASA, and other models. Aircraft and SFT differ greatly in tire size, load, pressure, etc., so there are also large differences in the available friction coefficients. The correlation model can convert the estimated value of the ground friction coefficient into the aircraft braking friction coefficient to predict the safety grade of the aircraft during braking. The method proposed in this paper aims to overcome the deficiencies of traditional measuring equipment and estimate the grade of the aircraft braking friction coefficient on the runway surface, even if there is no expensive experimental equipment.

This paper introduces a relative survey and research on the airport runway friction coefficient. The remainder of this paper is organized as follows: Section 2 presents the related works on measuring friction coefficient. Section 3 introduces related terms and discusses the factors affecting the runway friction coefficient. Section 4 minutely describes the measurements for the friction coefficient. These methods include the cause-based and the effect-based methods, an instrument for runway surface test, and aircraft dynamics model. Section 5 explains the feasibility, variable selection, and measurement principle of the multi-sensor information fusion scheme proposed in this paper. Section 6 summarizes the correlation model between the aircraft braking friction coefficient, and the ground friction coefficient and attempts to predict the grade of the aircraft braking friction coefficient through the correlation model. Section 7 discusses the advantages and disadvantages of these methods. Finally, the conclusion and future work are explained in Section 8.

## 2. Related Work

The earliest method for friction measurement is the standard test designed by the American Society for Testing Materials (ASTM), which is suitable for both roads and runways. At present, airports mainly use an SFT (or an airport surface friction tester, ASFT) or friction coefficient car (this term is more popular in China) to measure the runway friction coefficient. In response to the problems in the measurement process, China Civil Aviation University [3,4,5] and Hebei University of Technology [6,7] have given many solutions. Apart from these, different methods have been applied to estimate the tire–runway friction coefficient. These methods can be divided into two categories: the cause-based method and the effect-based method [8]. The cause-based method, also known as the experimental method, uses sensors to measure friction-related parameters and attempts to correlate these parameters with the tire–road friction coefficient. The friction-related parameters are to use a sensor to measure anything from hub sound to road surface optical properties, and these sensors are acoustic [9,10,11], optical [12,13,14,15,16], and tread sensors [17,18,19,20,21,22]. Meanwhile, the effect-based method attempts to simplify the mathematical models to estimate friction [23,24]. Therefore, tire models [25,26,27,28,29,30], vehicle dynamics models [23,31], and aircraft dynamics models [32,33,34,35,36,37] are often used to estimate the friction coefficient. However, the research of these algorithms is inseparable from the multi-sensor information fusion technology. The intelligent vehicle safety system (IVSS, a program launched by the Swedish) attempted to combine longitudinal and lateral forces with a road eye sensor [12]. Some Kalman-based filtering methods are also used as data fusion [38,39,40]. Further, it is essential to provide reliable runway information to the pilot when the aircraft is landing or taking off. However, aircraft and ground friction measurement devices (GFMD) have differences in tire size, pressure, speed, etc. The correlation between the ground friction coefficient and the aircraft braking friction coefficient has attracted significant attention from many researchers at home and abroad [41,42,43,44].

## 3. Tribo-System and Influencing Factors

**Figure 1:**
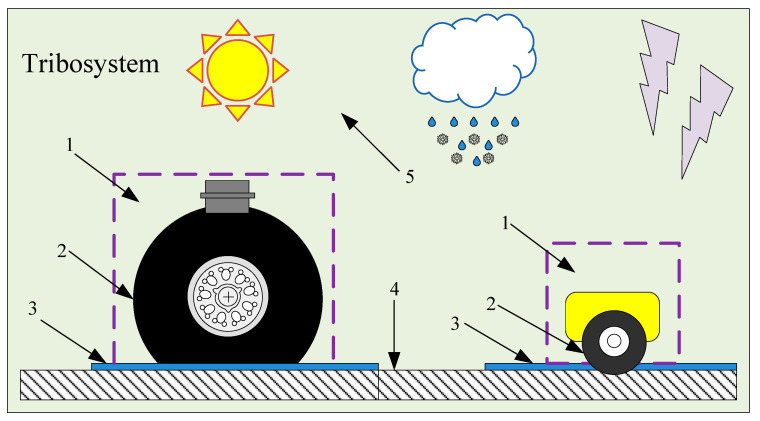
Sample tribo-system for braking slip friction: Aircraft landing gear braking on the runway surface and surface friction tester (SFT) on the runway surface. Where 1 is a tribometer, 2 is a tire, 3 is a deposit, 4 is a runway surface, and 5 is an ambient environment.

The interaction complexity of aircraft tires with runway surfaces is self-evident. Norheim, et al. [45] analyzed the interaction between them founded on the tribo-system and proposed a general, structured approach. Figure 1 shows two tribo-system models: an aircraft tire–runway model and an SFT tire–runway model. It consists of four tribo-components:

- Tires;
- Runway;
- Deposit;
- Atmosphere.

**Figure 2:**
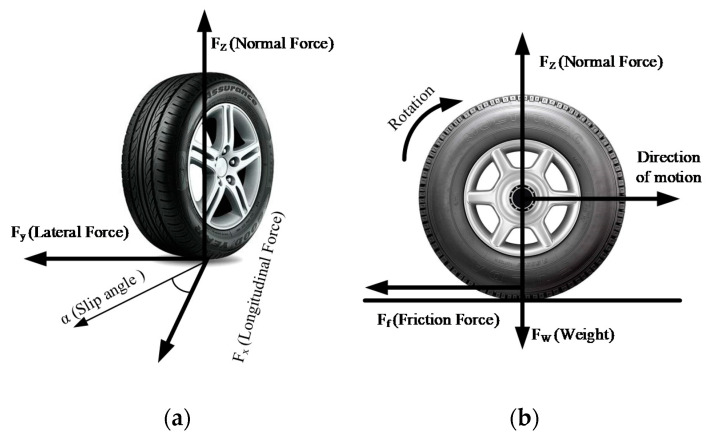
Force on a rotating tire: (**a**) free body diagram of a single wheel; (**b**) schematic diagram of longitudinal force.

Friction is a force that resists a relative movement between the tires and the runway. It enables the pilot to control the aircraft safely, either longitudinally or laterally. The forces are generated as shown in Figure 2 when the tire moves on the runway. It, characterized using the non-dimensional friction coefficient (μ), is the ratio of the tangential friction force ($F_{t}$) between the tire tread and the runway surface to the perpendicular force or vertical load ($F_{W}$).

(1) $$\mu =F_{t}/F_{W}$$

**Figure 3:**
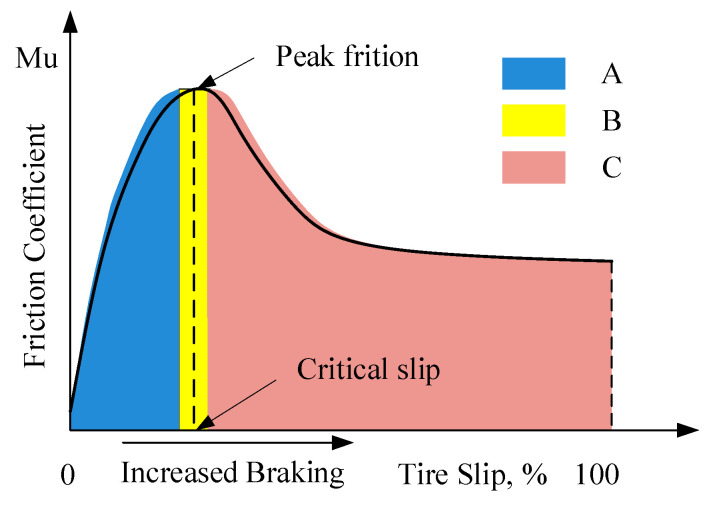
Relationship between the friction coefficient and the slip: **A** is the adhesion region, **B** is the desired region, and **C** is the slip region.

Longitudinal friction occurs when the tires are accelerating or braking. The relative speed between the tire and the runway (referred to as slip speed) is zero during free rolling, and the equations are

(2) $$v_{S}=v-v_{p}=v-(0.68\times \omega \times r)$$

(3) $$s=(v-v_{p})/v\times 100\%=v_{S}/v\times 100\%$$

where $v_{S}$ is the slip speed, v is the aircraft speed, ω is the tire angular velocity, r is the tire radius, s is the slip rate, and $v_{p}$ is the tire peripheral velocity. The friction coefficient between the tire and the runway surface varies with the slip rate, and the relationship between them has been obtained through experiments, as shown in Figure 3. Mahinder, et al. [46] has done a lot of literature investigations on the factors affecting the runway friction coefficient and obtained the conclusion shown in Table 1.

## 4. Measurement for Friction Coefficient

### 4.1. The Cause-Based Method

**Figure 4:**
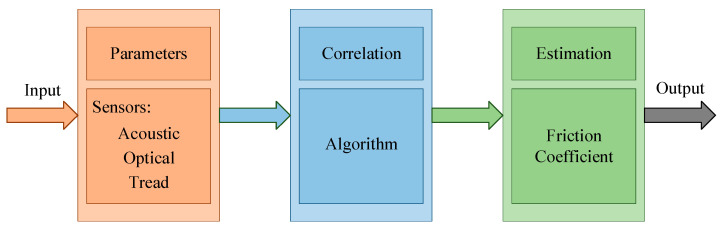
The process of an experimental method.

The cause-based method, also known as the experimental method, uses sensors to measure friction-related parameters and attempts to correlate these parameters with the tire–road friction coefficient, and its process is given in Figure 4. According to physical properties, these sensors can be divided into three types:

#### 4.1.1. Acoustic Sensor

**Figure 5:**
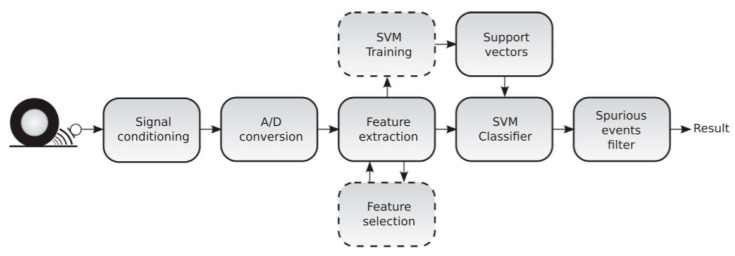
Principle diagram of friction estimation method based on acoustic sensor [9]. Reprinted with permission from [9].

The acoustic footprint of each road is different, and the noise frequency of the tire on the dry road is also distinct from that on wet. The road information can be understood by analyzing the tire–road noise. Alonso, et al. [9] proposed a real-time acoustic analysis system based on tire–road noise, using support vector machine (SVM) to classify the pavement state, which can identify the dry and wet state of asphalt pavement, and its principle diagram is shown in Figure 5. Kong [10,11] proposed a new method for automatically detecting the road surface state from tire noise, including dry, wet, snow, and mud, based on wavelet analysis, artificial neural network, and mathematical evidence theory. The following year, a method based on the frequency domain and time domain was proposed to classify the state of the pavement.

#### 4.1.2. Optical Sensor

**Figure 6:**
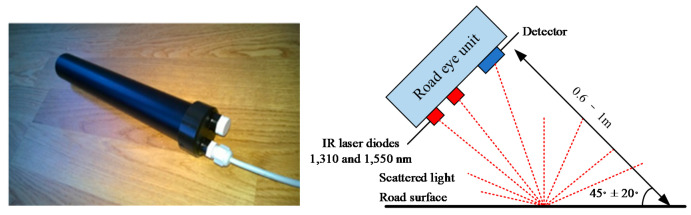
Road eyes (**left**) and the schematic of using an optical sensor to identify different surfaces (**right**) [23]. Reprinted with permission from [23].

The pavement texture and condition can reflect infrared light of different wavelengths, combined with cameras and optical sensors to detect friction-related pavement features. IVSS [12] proposed an optical sensor called road eye, as shown in Figure 6, which has the potential to classify different phases of water (ice, snow, and mixtures) because of its different resonance frequencies at near-infrared wavelengths (1–2 μm). Vikki [13] used non-contact 3D field technology to obtain the macrotexture of the pavement, and the measuring results showed a positive correlation with tire–road friction. Holzmann [14] used cameras to take pictures of the current environment, extracted different μ-corresponding patterns based on the overall brightness, and matched these patterns with the current environment to derive the friction coefficient and confidence of the road ahead, while the microphone improved the reliability. Sohini [15] proposed a two-stage method for indirect tire–road friction coefficient estimation by using camera images. In the first stage, three variants of Neural network (NN)-based models were used to learn the characteristics of a specific region, and in the second stage, the road surface was divided into 5 × 3 blocks for quantization.

The development of optical sensors has come further, in particular on the classification of snow, ice, and water on pavement. At present, some commercially available sensors have been widely used. Most of these sensors can provide pavement temperature, subsurface temperature, pavement conditions (dry, wet, frozen, etc.), chemical snow remover residues, etc. Some sensors can also measure black ice, dew point temperature, and other related pavement parameters. Their detailed features are shown in Table 2.

#### 4.1.3. Tire Tread Sensor

**Figure 7:**
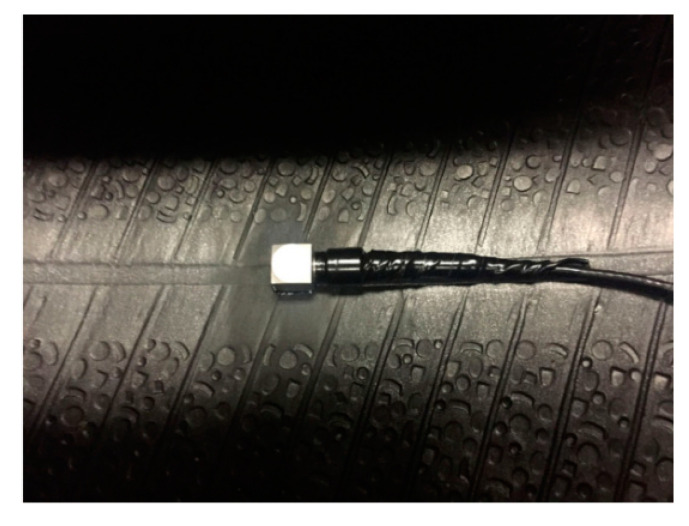
Tri-axial accelerometer tread sensor [23]. Reprinted with permission from [23].

The tire tread sensor, embedded on the surface of the inner tire, is used to monitor the interaction between the tire and the road surface, and to estimate the friction characteristics between them. The main types are piezoelectric, acoustic surface, and optical sensors. A tri-axial accelerometer tread sensor is shown in Figure 7. Arto [17] used three three-axis accelerometers as tire sensors to estimate friction based on acceleration signals. Zhang, et al. [18] installed passive wireless acoustic surface sensors inside the tires to achieve the function of automatically measuring tire pressure and temperature parameters. Pohl, et al. [19] estimated the tire–road friction coefficient based on the strain of the tread element. Tuononen, et al. [20] developed a tire sensor based on optical position detection. It can measure the deflection of the carcass relative to the rim, which can be used to calculate the tire force (vertical force and lateral force) and to estimate the friction coefficient. Both Yi [21] and Erdogan [22] measured the parameters related to friction through the tread sensor.

### 4.2. The Effect-Based Method

The effect-based method is a model method. For the measurement of the airport runway friction coefficient, there are two commonly used model methods as follows. Of course, the car model can also be regarded as a method in this field.

#### 4.2.1. Tire Model

The tire model can be divided into three types: theoretical model, empirical model, and semi-empirical model. Each model contains a variety of specific tire models. The tire model used in this paper is the Pacejka model, also known as the magic formula tire model (MFTM), which is a model based on the longitudinal force, lateral force, and aligning torque of a tire [25] and is widely used in research.

(4) $$\begin{array}{l} F_{x}(s+S_{hx})=D_{x}\sin\left\{C_{x}\arctan\left[B_{x}s-E_{x}(B_{x}s-\arctan B_{x}s)\right]\right\}+S_{vx}\\ F_{y}(\alpha +S_{hy})=D_{y}\sin\left\{C_{y}\arctan\left[B_{y}\alpha -E_{y}(B_{y}\alpha -\arctan B_{y}\alpha )\right]\right\}+S_{vy}\\ M_{z}(\alpha +S_{hz})=D_{z}\sin\left\{C_{z}\arctan\left[B_{z}\alpha -E_{z}(B_{z}\alpha -\arctan B_{z}\alpha )\right]\right\}+S_{vz} \end{array}$$

There is no noteworthy relationship between the longitudinal force and friction coefficient in Equation (4). Kyongsu [26] used the MFTM to estimate the tire–road friction coefficient (Equation (5)). Germann [27] proposed an equation to describe the friction coefficient and slip based on the MFTM (Equation (6)). Alberto [28] used the model approximation method of Jacobi polynomial series expansion magic formula to estimate tire force Equation (7). Rui, et al. [29] and Zhun, et al. [30] gave the relationship between model parameters and tire pressure and load.

(5) $$F_{x}=F_{x}(F_{z},s,\mu )=\mu F_{x_{0}}(F_{z},s,\mu_{0})$$

(6) $$\mu =D\sin\left\{C\arctan\left[B(1-E)(s+S_{h})+E\arctan\left(B(s+S_{h})\right)/B\right]\right\}$$

(7) $$F=A_{0}+A_{1}[x/(x+b)]+A_{2}{\left[x/(x+b)\right]}^{2}+A_{3}{\left[x/(x+b)\right]}^{3}$$

where *F* is the lateral force or longitudinal force (for self-aligning torque, a fourth-degree polynomial should be used to obtain a better accuracy); *x* is the longitudinal slip or slip angle, and *Ai* and *b* are the basic parameters of the model.

#### 4.2.2. Aircraft Model

Aircraft braking depends on the friction force between the tire and the runway, which is affected by many factors, such as slip rate, speed, tire type, and runway surface condition, and so on. Essentially, these factors have an impact on the friction coefficient between the braking tire and the runway surface [47]. The wheels are decelerated by the braking torque, the tires slide relative to the runway, and the resulting friction is the braking force.

**Figure 8:**
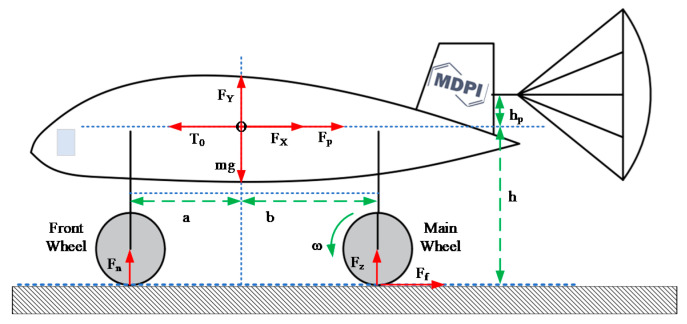
Analysis of the forces on aircraft ground taxiing.

Two kinds of aircraft ground kinematics equations were established. One was to simplify the aircraft motion to a linear motion model with a single wheel and a part of the aircraft mass [32,33]. The other was to consider the force of the aircraft in the taxiing process and regarded other secondary factors as disturbances to the system [34,35]. Force analysis of aircraft taxiing is shown in Figure 8, and the meanings of these symbols shown in Figure 8 are given in Appendix A. In establishing the aircraft ground kinematics equations, some assumptions are made, Fu [34] describes them in detail. The aircraft longitudinal motion equation (Equation (8)), aircraft vertical force balance equation (Equation (9)), torque balance equation (Equation (10)), and braking wheel dynamics equation (Equation (11)), were established.

(8) $$m\dot{v}+F_{X}+F_{p}+nF_{f}-T_{0}=0$$

(9) $$F_{Y}+F_{n}+nF_{Z}-G=0$$

(10) $$nbF_{Z}+h_{p}F_{p}-nhF_{f}-aF_{n}-T_{0}h_{p}=0$$

(11) $$J_{\omega }\dot{\omega }=F_{f}R-B_{w}\omega -T_{b}$$

(12) $$T_{b}=0.5\mu_{f}n_{f}(r_{d}+r_{s})P_{A}=K_{b}P_{A}$$

Boeing has developed an aircraft braking performance model, which is used to determine the aircraft braking coefficient and this model is used to establish a motion equation as follows:

(13) $$\begin{array}{l} m\cdot \frac{dv}{dt}=D_{thrust}-D_{aero}-mg\sin\varepsilon -D_{brakes}\\ D_{brakes}=\mu_{B}(mg\cos\varepsilon -L) \end{array}$$

where g is the gravitational constant, dv/dt is the acceleration, $D_{thrust}\;$ is the force caused by thrust, $D_{aero}$ is the aerodynamic drag, ε is the runway slope, $D_{brakes}$ is the force contribution from the wheels, L is the aerodynamic lift, and $\mu_{B}$ is the effective aircraft braking coefficient. Boeing uses $\mu_{B}$ to denote the contribution of aircraft wheel braking to aircraft stopping, including the effects of wheel braking and contaminated resistance. In some cases, it reflects the amount of tire–road friction used [36]. Further, Boeing provided the aircraft landing distance as a function of braking action. To be able to directly compare with the runway surface evaluation, $\mu_{B}$ and the friction coefficient measured from the GFMD are used to explain it, as shown in Table 3. 

Airbus [37], another large transport aircraft manufacturer, has also developed a model for estimating the used wheel braking friction of an in-service commercial aircraft. Airbus and its subsidiary NAVBLUE have developed a new technology to use the aircraft itself as a sensor to measure the available runway braking action. Until 2018, Airbus developed a new aircraft function, and the implementation of this function on an Airbus aircraft is called the “braking action computation function (BACF)”. The fundamental principle of the function is, post-landing, to use the data measured by the aircraft during its deceleration roll to identify the braking action level. By using the aircraft performance model, it is possible to differentiate the part of deceleration coming from either aerodynamic, thrust reverse, or wheel-braking. Both Boeing and Airbus use engine models to calculate the reverse thrust contribution, which are proprietary models that include both the effect of crosswind, reversed engine thrust, and runway slope.

### 4.3. Airport Runway Friction Coefficient Test Car 

To ensure the safety of the aircraft take-off and landing process, the runway friction coefficient must be measured by using an airport runway friction coefficient test car (also called a surface friction tester, SFT) when necessary, especially in the frequent takeoffs and landings, or when there are rain and snow deposits on the runway surface. ICAO stipulates that the frictional characteristics of the runway surface must be determined regularly by using a continuous friction tester equipped with a self-wetting device. 

**Figure 9:**
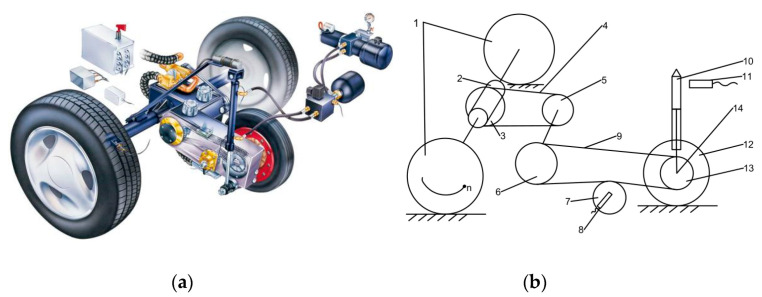
Rear axle system of SFT: (**a**) real rear axle system; (**b**) simplified rear axle system. Where, 1—vehicle rear wheel, 2—unidirectional differential, 3—first active sprocket, 4—first transmission chain, 5—first passive sprocket, 6—second active sprocket, 7—tensioner, 8—horizontal force sensor, 9—second transmission chain, 10—hydraulic cylinder, 11—take-up relay, 12—measuring wheel, 13—second passive sprocket, 14—vertical force sensor.

**Figure 10:**
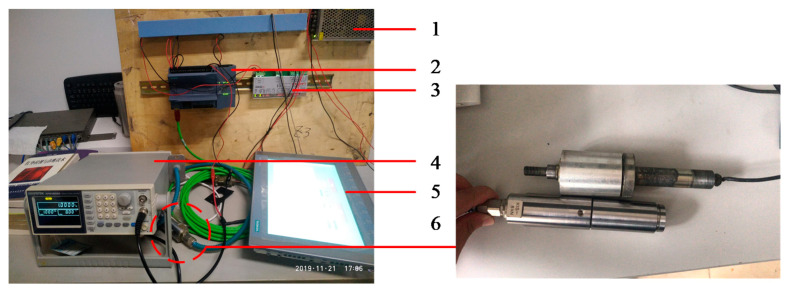
Debugging system in the laboratory, where 1 is a power system; 2 is a programmable logic controller (PLC); 3 is a load cell signal transmitter; 4 is a signal generator, which is used to simulate the speed sensor; 5 is touch a screen; 6 is a horizontal force sensor. Communication between 2 and 5 via industrial ethernet (green cable).

**Figure 11:**
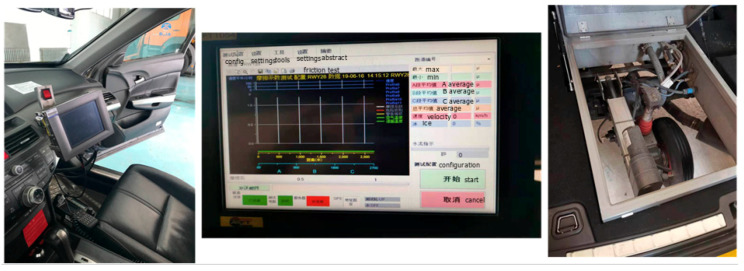
Debugging system on-site, touch screen in co-pilot position (**left**), real-time friction coefficient curve on the touch screen (**middle**), and SFT’s rear axle system, located in the rear compartment (**right**).

It is an electromechanical-hydraulic integrated high-tech aviation ground equipment composed of a mechanical transmission system, hydraulic system, computer measurement and control system, and electrical control system. It can be used for civil and military airport runways, as well as for highways, and the reliability of its measuring result has important practical significance for the safety of aircraft, traffic, and road. To achieve the measuring function, manufacturers designed a dedicated rear axle system that is given in Figure 9 and it adopts a fixed-slip measurement. It is composed of a one-way clutch, chain transmission gear, multi-row transmission chain, and other structures, which makes it have the function of differential speed and power transmission. When the rear wheels (1) rotate, they drive the unidirectional differential (2) to rotate, which is driven by the active sprocket, the passive sprocket, and the transmission chain, and finally, drive the measuring wheel (12) to rotate. During the rotation of the measuring wheel, the friction with the ground acts on the tensioner (7) through the transmission chain (9), and the tensioner is equipped with a horizontal force sensor (8), which can measure the force applied to the tensioner. Figure 10 is the electrical part of the debugging system in our laboratory, and Figure 11 is the test system on-site.

**Figure 12:**
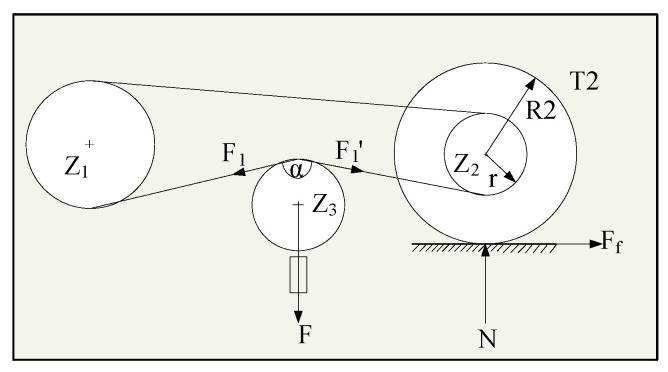
Schematic diagram of the SFT calculation principle.

The measuring speed is 96 km/h. Under the action of the hydraulic system, the measuring wheel generates a pressure of 140 kg against the ground. The friction ($F_{f}$) between the measuring wheel and the runway surface forms an equal and opposite direction force on the transmission chain through the action of torque, and the resultant force (F) can be obtained by calculation. Then, the calculation schematic for SFT is shown in Figure 12.

$F_{1}$ can be obtained from the torque balance:

(14) $$F_{1}=F_{1}^{\prime }=F_{f}R_{2}/r$$

The force of the horizontal sensor can be obtained from the cosine theorem:

(15) $$\begin{array}{ll} F & =\sqrt{2F_{1}^{2}-2F_{1}^{2}\cos(\pi -\alpha )}\\ & =\sqrt{2}\sqrt{1+\cos\alpha }F_{1}\\ & =\sqrt{2}F_{f}R_{2}\sqrt{1+\cos\alpha }/r \end{array}$$

The expression of friction is obtained from Equation (15):

(16) $$F_{f}=Fr/(R_{2}\sqrt{2(1+\cos\alpha )})=kF$$

Meanwhile, the expression of the friction coefficient is

(17) $$\mu =F_{f}/F_{N}\approx 7.29\times 10^{-4}kF$$

## 5. Friction Coefficient Estimation Based on Multi-Sensor Information Fusion 

### 5.1. Feasibility Analysis Based on Multi-Sensor Information Fusion 

**Figure 13:**
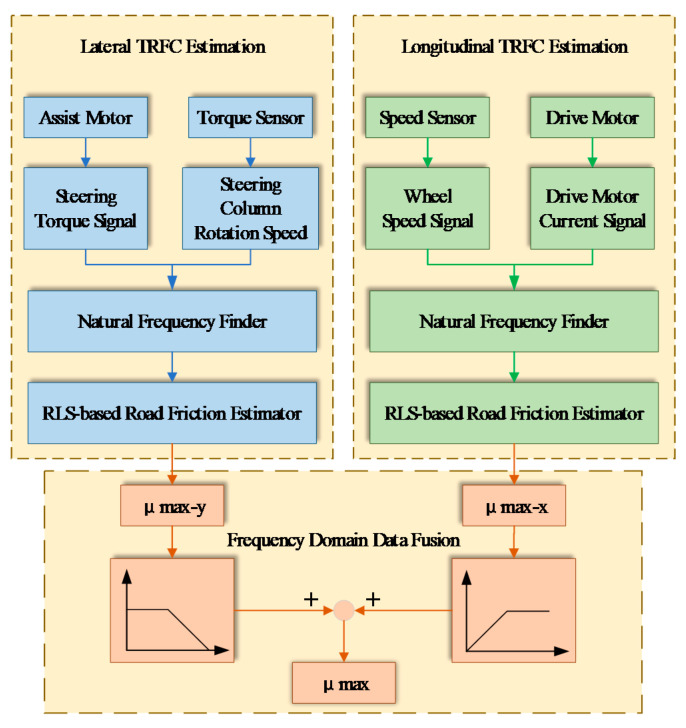
Friction coefficient measurement: process of the estimation method. Reprinted with permission from [40].

Right now, multi-sensor information fusion technology is widely used in various fields, meanwhile, it has also made a great contribution to the friction coefficient measurement. For example, IVSS [12] proposed a combination of the force-based and indirect optical methods. The longitudinal force was obtained when accelerating or braking, and the lateral force was obtained when turning. Considering these two force-based algorithms as independent, using a third fusion algorithm will improve the friction estimation performance and usability. For improving the tire–road friction coefficient estimation, Bin, et al. [38] used an adaptive extended Kalman filter based on finite memory, and Liu, et al. [39] used the combination of an auxiliary particle filter and iterative extended Kalman filter. The tire–road friction coefficient estimation based on frequency domain data fusion was proposed by Chen, et al. [40], and its fusion process is given in Figure 13.

### 5.2. Variable Analysis and Sensor System

Multi-sensor information fusion technology is more and more widely used in the friction coefficient estimation. Section 3 summarizes the existing methods, and it is not difficult to find that the cause-based method uses various sensors to obtain the runway surface conditions. For the effect-based method, the tire model can be regarded as a special sensor and the friction coefficient between the tire and the road surface can be obtained through an algorithm. Mahinder [46] repeated the analysis of the variables in Table 1 and found that the main factors affecting the friction coefficient are: tire width, tread depth, outside diameter, inflation pressure, vertical load, fluid depth and density, peak available mu, runway macrotexture, and forward ground speed. 

Acoustic sensors’ contribution to the friction coefficient measurement is to classify runway contaminants according to their acoustic footprint, which can identify the four states of dry, wet, snow, and mud. Optical sensors not only can classify runway contaminants but also can measure their density and thickness through light reflection and refraction. The contaminants on runway surfaces including slush, frost, ice, and snow can be divided into dry snow, wet snow, and compacted snow. The difference among them is the density. Therefore, three tasks can be completed by optical sensors: 1. combining with acoustic sensors to accurately classify contaminants; 2. measuring the density of contaminants; and 3. laser texture instruments can measure the runway surface texture. Further, tread sensors can be used to measure tire load, pressure, and deceleration when the car is braking. It can completely replace the vertical force sensor of the SFT to monitor tire load in real-time, and the cost is much lower than it. As for the tire model, the relevant literature has given the parameters of MFTM, which can be calculated by Equation (6) or (7). The friction coefficient calculated by these formulas is compared with those measured with a horizontal force sensor and estimated based on multi-sensor information fusion. 

The above discussion is the input variables for the sensor system. After analysis, the input and output variables as a multi-sensor information fusion model are shown in Table 4. The output variable $y_{1}$ is the friction coefficient measured by using SFT, and $y_{2}$ is the friction coefficient estimated based on multi-sensor information fusion. It is worth noting that $x_{10}$ can be used as both input and output. The purpose of an input is to improve the estimation accuracy through MFTM, while as output is to compare with $y_{2}$ and modify the model. 

### 5.3. Fusion Algorithm, Data Acquisition, and Real-Time Calculation

So far, fusion algorithms can be categorized into four types: estimation algorithm, parameter algorithm, recognition algorithm, and artificial intelligence algorithm. The variables in Table 4 belong to different types and the neural network model in the artificial intelligence method can highly correlate these unrelated variables. This paper selects a neural network as the algorithm for multi-sensor information fusion. Therefore, the runway friction coefficient estimation is inevitably divided into three stages.

The first stage is data acquisition that is used to train the neural network. The data acquisition should include $x_{1}\sim x_{10}$ and $y_{1}$. Data collection is inseparable from an SFT in that its core sensing components are the sensors used to measure forces: horizontal force sensor and vertical force sensor. The vertical force sensor is used to measure the wheel load. The tread sensor can replace it in this paper and can reduce the cost. Meanwhile, the horizontal force sensor is used to measure the overall force and obtain the friction between the tire and runway by calculation. These calculations are done by the SFT’s host computer (PLC) and displayed on the slave computer (touch screen) in the form of a friction coefficient. These sensors, which are used to measure $x_{1}\sim x_{10}$, are not so much integrated as a sensor system, but rather a mobile weather–runway–tire system because they are installed on an SFT. It is worth noting that since $x_{1}$ contains four states, the data in each state need to be included in this neural network model.

The second stage is network training. Through training, the relationship between different types of sensors and friction coefficient can be obtained.

**Figure 14:**
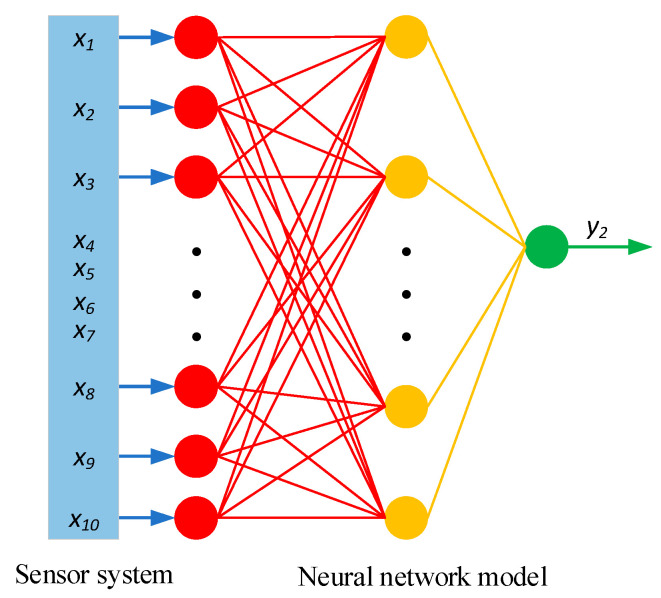
A neural network model proposed in this paper based on multi-sensor information fusion.

The third stage is a real-time calculation. Although SFT is the most widely used system for measuring the airport runway friction coefficient, based on the fact that it is a specialized device that has been transformed, the rear axle system has a complex structure, and the core sensors—horizontal force sensor and vertical force sensor—require regular calibration; meanwhile, it often brings troubles during the measuring process. The inputs are used to train the neural network by using sensors to measure the parameters related to the runway surfaces and contaminants, while the output is the friction coefficient calculated by the SFT’s horizontal force sensor. In the second stage, the model between the inputs and output has been obtained. The principle of real-time calculation is that it only needs to provide inputs to the neural network and it will automatically get the corresponding output. Therefore, this paper proposes a method based on multi-sensor information fusion, where the essence is to use a sensor system to replace the SFT rear axle system. Figure 14 shows the estimation method proposed in this paper.

## 6. Correlation of Ground Friction Measurements to Aircraft Braking Friction

From 1996 to 2003, the United States, Canada, and Europe jointly launched a project called the “Joint Winter Runway Friction Measurement Program” (JWRFMP) that focused on the ability of aircraft tires to interact with the runways in winter to provide sufficient wheel braking for the landing and acceleration-stop operation. Taking the GFMDs, the perceptual report of the pilot’s braking action and the description of the contaminants on the runway surface (especially in winter), as information sources, the Aviation Rulemaking Committee for Takeoff and Landing Performance Assessment (TALPA) established a runway condition assessment matrix (RCAM) [44]. Many researchers [45,46,47] studied the maneuverability of the aircraft under severe weather conditions, determined the relationship between the aircrafts and the GFMDs, and several correlation models were given.

### 6.1. Kollerud Theory

The relationship between the aircraft braking performance and GFMD on contaminated runways in winter has been studied for many years. Kollerud theory, the first approved method, uses the complete stop method to determine that the aircraft effective friction coefficient is half of the measured friction coefficient.

### 6.2. IRFI and CRFI model

JWRFMP established a large database on the data of 47 kinds of GFMDs and developed a coordination program of the IRFI through statistical analysis of their linear correlation. Another test on five types of test aircrafts under snow and ice-covered runway surface conditions was conducted based on experimental data from 1998 to 2001 to get a correlation model where the IRFI values were derived from both the IMAG trailer (runway surface tester with adjustable slip rate, 15% slip) and the electronic recording deccelerometer (ERD). In addition to the above two devices, other GFMDs have been verified, including SAAB 95 (predecessor of ASFT), and $R^{2}=0.78$.

(18) $$\left\{ \begin{array}{l} \mu_{Brak\_IRFI}^{AC}=0.751\times IRFI-0.056\\ R^{2}=0.85 \end{array}\right.$$

Canada also proposed the CRFI and obtained the equation between the aircraft braking coefficient and CRFI, but it is only valid for one particular friction measurement device, the ERD.

(19) $$\left\{ \begin{array}{l} \mu_{Brak\_CRFI}^{AC}=0.40\times CRFI+0.02\\ R^{2}=0.89 \end{array}\right.$$

According to the IRFI calculated by standard friction measurement, the relationship between ground friction and the aircraft braking coefficient is expressed as

(20) $$\left\{ \begin{array}{l} \mu_{aircraft}=0.41\times \mu_{ground}+0.03\\ R^{2}=0.64 \end{array}\right.$$

Later, New Chitose Airport used the Saab friction tester to correlate with the aircraft braking friction coefficient, and got the following expression:

(21) $$\left\{ \begin{array}{l} \mu_{Brak\_Saab}^{AC}=1.1568\mu_{Saab}-0.2043\\ R^{2}=0.886 \end{array}\right.$$

### 6.3. NASA Model

NASA Research Center has been devoted to this research since 1973. The Federal Aviation Administration ((FAA) conducted a large number of tests using B727 and B737, 12 runways at 6 locations, 5 surface conditions (dry, wet, snow, mud, and ice), and 6 ground friction coefficient cars, and obtained the friction coefficient relationship between aircraft braking and GFMDs as

(22) $$\mu_{B\mathrm{rak}\;\mathrm{ABS}}^{AC}=0.2\times \mu_{ground}+0.7143\times \mu_{ground}^{2}$$

**Figure 15:**
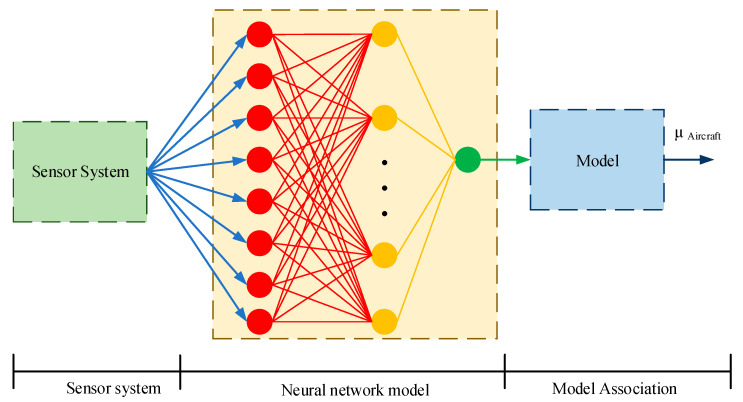
Estimation for runway friction coefficient based on multi-sensor information fusion and model correlation.

Combined with multi-sensor information fusion, the proposed scheme in this paper is shown in Figure 15.

## 7. Discussion

Reviewing the previous sections, a total of five methods that can be used for measuring the runway friction coefficient are proposed in this paper, and they are:

- ASTM standard methods;
- Friction coefficient car;
- The cause-based;
- The effect-based;
- Multi-sensor information fusion and model correlation.

For each method mentioned above, this section discusses them in detail. 

ASTM standard methods are the earliest method proposed to be applied to measure the road friction coefficient. SFT itself is one of them, but this paper makes it independent of them. Therefore, the methods include braking test, contact test, and non-contact test. The measuring accuracy of the braking test is low, and the tire wear is great. The contact test uses portable instruments, such as the British pendulum tester and dynamic friction tester, so that the time-consuming measuring results only represent a small part of the area under test and traffic must be regulated during the measurement. Regardless of accuracy or repeatability, this method does not meet the needs of today’s transportation industry. However, the laser-based method, a high-speed laser texture measuring device with high accuracy, belongs to a non-contact test that uses ultra-high frequency laser triangulation sensors to measure the macrotexture of the runway surface.

The measuring accuracy and repeatability of the friction coefficient car ensure that it is a device widely used by all airports, and it can continuously measure on the runway. Even so, it has many problems, which mainly reflect in the unreasonable mechanical structure, complicated hydraulic system, and one-way differential transmission that restricts driving. The rear axle system, with a total weight of 700 kg, has a complicated structure and is difficult to manufacture and has a great impact on acceleration and safety. When the car is driving in a non-measurement state, the one-way differential transmission system will be continuously driven to work, which affects the service life of the entire system. Especially when the measuring wheel is in contact with the ground. If the car turns or reverses, it will cause big damage to the one-way differential transmission system. Frequent tire replacement and repeat calibration sensors also increase the workload of employees.

The related literature [8,10,11,15] showed that the contribution of the caused-based method to measure the friction coefficient is not outstanding. It obtains the parameters related to friction, not the friction itself. Ref.10 and 11 classify the types of contaminants by the acoustic footprint of the tire and the road surface, and the result is only the road surface conditions. Ref.15 quantizes the divided blocks based on optics and finally as only high, medium, and low to indicate the friction coefficient in each block. Ref.23 summarizes the accuracy and repeatability of each sensor-based method.

With the increase in traffic demand, tire mechanical properties have been extensively studied. In addition to the MFTM proposed in this paper, they are commonly used brush models, LuGre models, etc. The advantage of these models is that they distinguish between different working states and even different contaminants. However, these models have a large number of parameters, which are different under different pavement materials. Before using, a lot of experiments for fitting parameters needs to be done. Due to its particularity, the accuracy of the tire model is relatively high. The aircraft model also belongs to the category of the effect-based methods. However, without expensive experimental equipment, this method only stays in theoretical research. Further, in the analysis process, many influencing factors are ignored, so the accuracy of this method is open to question. Unlike the IRFI, CRFI, and NASA models, they are based on Boeing 727, 737, and GFMDs to make a large number of experiments and obtain the correlation model through regression analysis. In recent years, Boeing and Airbus have established aircraft braking models, although these models have not been made public. Boeing uses $\mu_{B}$ to denote the contribution of aircraft wheel braking to aircraft stop, including the effects of wheel braking and contaminated resistance. Airbus has developed a new technology which is called braking action computation function (BACF) in order to use the aircraft itself as a sensor to measure the available runway braking action.

The main purpose of this paper is to estimate the runway friction coefficient based on the friction-related parameters measured by sensors. The methods proposed in this paper applied to runway friction measurement have some defects. Combining their merits, a method based on multi-sensor information fusion is proposed to measure the runway friction coefficient. The method is divided into three stages.

- In the first stage, the sensor system is built, and it is installed on a friction coefficient car to collect data;
- In the second stage, the network is trained, and the friction-related parameters and the friction coefficient are obtained through the neural network;
- The third is a real-time estimation, the sensor system collects data in real-time, and automatically obtains the real-time friction coefficient through the neural network model.

The friction coefficient car, a kind of special equipment that has been modified, is widely used in airports. Equations (14)–(17) give the calculation method of the friction coefficient between the tire and ground. Our research group is developing a new friction coefficient car and Figure 10 and Figure 11 are the debugging processes. The model derived from the method described in this paper can substantially replace the rear axle measurement system of the friction coefficient car. By comparison, the advantages of this method can be drawn.

- Compared with ASTM standard methods;
- The sensor system is installed on the car, and can measure the friction coefficient of any position in the entire runway without traffic control, and can repeat the measurement;
- Compared with the friction coefficient car;
- Under winter conditions, the friction coefficient car changes tires on average every 10 days. Once the original is replaced, its core sensors need to be re-calibrated. The sensor system proposed in this paper can replace the core sensors without re-calibration and greatly reduce costs;
- Compared with the cause-based and the effect-based method;
- Friction is the result of the joint action of the tire and the runway. The cause-based method uses sensors to measure the parameters related to the runway surface, while the tire-based method pays more attention to the characteristics of the tire itself. In the sensor system, the optical sensor is used to measure the surface characteristics of the runway, and the tread sensor is used to measure the characteristics of the tire. The combined effect of the two is considered to make the prediction more accurate.

Although the sensor system can replace the measurement system of the friction coefficient car, it is still inseparable from the data collection process. In theory, when the relationship model between the inputs of the sensor system and the friction coefficient is obtained, the sensor system can be installed on an ordinary car to measure the friction coefficient, and the existence of the friction coefficient car becomes meaningless. However, in fact, it is not. The friction coefficient measured by the friction coefficient car is $y_{1}$, and the friction coefficient estimated by the neural network is $y_{2}$, assume the following equation:

(23) $$y_{1}=my_{2}+n$$

where m and n are the fitting coefficients, the purpose of Equation (23) is to obtain a linear expression between $y_{1}$ and $y_{2}$ by fitting, to minimize the error between the two. The equation for the final estimated friction coefficient is

(24) $$\mu_{estimation}=my_{2}+n$$

Airbus pointed out that the airport runway friction assessment can be performed using a variety of equipment and vehicles, which are based on the same measurement principles and experimental methods. The correlation between the data they generate and aircraft performance is challenged by factors such as the size and inflation pressure of the test wheel, the load on the test wheel, and the test speed. Therefore, airport runway friction assessment should be viewed as a way to monitor trends, rather than determine absolute values. Aircraft landing performance is not only related to the runway friction coefficient but also the operator’s operating procedures. This is why TALPA uses the runway friction coefficient as a secondary factor when establishing RCAM. Further, this paper proposes a correlation model method to predict the grade of the aircraft braking coefficient. Although the research on the relationship between the aircraft braking friction coefficient and ground friction is too scattered, several models can be used as a reference. The ground friction coefficient predicted by the neural network can be used to obtain the grade of the aircraft brake friction coefficient through the correlation model.

## 8. Conclusions and Future Work

This paper proposes a scheme of runway friction coefficient estimation based on multi-sensor information fusion and a correlation model to predict the grade of the aircraft braking friction coefficient. At present, there are few studies on airport runway friction, but for road friction studies, people have proposed the cause-based method—measuring parameters related to road friction—and the effect-based method—measuring the force generated by tire deformation to calculate the friction coefficient. These methods have been proven to be accurate and widely used. However, friction is the result of the joint action of the tire and the road surface, and they all seem to ignore the other party’s contribution to the friction. The sensor system proposed in this paper takes into account both the runway surface conditions and the tire conditions, and the friction coefficient is estimated under the joint action of the two.

However, there are some problems with the method proposed in this paper. For example, before training a model, a large amount of data needs to be collected. Further, whether the sensor system works at the airport will be affected by radio waves, and these factors need to be taken into account. Moreover, in future work, due to commercial optical sensors, the input information presented in Table 4 can be measured. Therefore, some experiments should be considered to prove the reliability of the method proposed in this paper. Further, as many as 48 factors affecting friction are listed in Table 1. In the process of measuring runway friction with SFT, other factors should be fully considered. Related studies show that the tread temperature of high-speed tires increases due to rolling resistance, which has a certain impact on the friction coefficient. The primary task of the method of estimating the friction coefficient based on multi-sensor information fusion is to find the factors that make the greatest contribution to friction, meanwhile, the measurement should be continuously improved. The new measurement should have the following properties: 1. must be able to continuously measure the runway friction performance; 2. with lower maintenance cost and equipment cost; 3. no traffic control and runway closure; and 4. it is applicable under all conditions.

## Figures and Tables

**Table 1:** Related factors affecting the friction coefficient.

Category	Detailed	Category	Detailed
Tire	1. Tire load	Pavement	1. Type
	2. Tire inflation		2. Surface texture
	3. Tire size		3. Microtexture
	4. Tire construction and design		4. Macrotexture
	5. Tire tread pattern design		5. Resistance to polishing by traffic
	6.Chemical formulation		6. Resistance to abrasion and crushing strength
	7. Polymer type		7. Weathering characteristics
	8. Carbon black type		8. Temperature
	9. Curing system		9. Thermal properties
	10. Other ingredients of tread compound		10. Matrix properties
	11. Physical properties		11. Contamination
	12.Tread surface conditions		12. Grooving
	13. Surface degradation	Operating Conditions	1. Traffic density
	14. Chemical/physical absorption		2. Velocity
	15. Thermal properties		3. Tire slip, peak or locked-wheel conditions
	16. Dynamic properties		4. Site design
	17. Surface temperature		5. Prevailing climatic conditions
Lubricant	1. Type		6. Testing vehicle design
	2. Viscosity		7. Method of measurement
	3. Surface tension		8. Stopping distance
	4. Film depth		9. Decelerometer
	5. Film strength		10. Cornering force coefficient
	6. Temperature		11. Braking force coefficient
	7. Impurities		12. Towed vehicle (impending slide)

**Table 2:** Commercial optical sensors.

Nation/Company	Product	Feature
Finland	RCM411 RTS411	It is a vehicle-mounted road surface condition rapid detection device, which can detect the road surface status in real-time, and is mainly used to provide a decision-making basis for road maintenance in winter. The device is also suitable for testing airport runways. RCM411 can detect all road conditions, including dry (green light), wet (light blue), accumulated water (dark blue), muddy (purple), snow (white), and icy (red). In addition to the surface condition, the output information includes friction coefficient, water film thickness, and optional surface temperature sensor RTS411.
Germany/ LUFFT	MARVIS	The mobile road weather sensor MARVIS turns vehicles into driving weather stations by detecting several critical road and runway weather parameters. Further, it can deliver information about temperatures, water film thickness, dew points, road conditions (dry, moist, wet, snow, ice), ice percentages, real humidity, and friction.
Sweden/ Met Sense	MetRoad Mobile	MetRoad Mobile is a mobile laser sensor for road conditions and road friction detection. Based on near-infrared spectroscopy, it can identify other unknown road conditions except for dry, moist, wet, ice, slush, and snow. It can measure the dimensionless friction coefficient from 0 to 1.
Finland/Vaisala	DRS511	It not only provides the temperature of the surface, but it also detects the presence of moisture on the surface, and thus provides a road state such as dry, wet, ice, and snow.
	DSC111	Several measurements combined compactly in one sensor. It can individually identify the presence of water, ice, slush, snow, or frost, and accurate and stable measurement results even with intense traffic.

**Table 3:** The grade of the aircraft braking coefficient and ground friction coefficient.

Code	Braking Coefficient	Friction Coefficient
5.Good	$\mu_{B}>0.2$	0.40 and above
4.Medium-Good	$0.2\geq \mu_{B}>0.15$	0.39 to 0.36
3.Medium	$0.15\geq \mu_{B}>0.10$	0.35 to 0.30
2.Poor-Medium	$0.10\geq \mu_{B}>0.075$	0.29 to 0.26
1.Poor	$0.075\geq \mu_{B}>0.05$	0.25 and below
0. NIL	$0.05\geq \mu_{B}$	/

**Table 4:** Input and output of the sensor system proposed in the paper.

Type	Category	Symbol	Variable
Input	Optical sensor	$x_{1}$	Runway condition
		$x_{2}$	Runway contaminant density
		$x_{3}$	Runway contaminant depth
		$x_{4}$	Runway surface temperature
		$x_{5}$	Runway surface texture
	Tread Sensor	$x_{6}$	Tire pressure
		$x_{7}$	Tire load
		$x_{8}$	Deceleration
	Others	$x_{9}$	SFT forward speed
Input/Output	Tire model	$x_{10}$	Friction coefficient based on magic formula
Output	Force sensor	$y_{1}$	Friction coefficient measured by SFT
	Estimator*	$y_{2}$	Friction coefficient estimated by estimator

* Estimator represents the neural network model shown in Figure 14.

## Appendix A

Nomenclature			
μ	Dimensionless friction coefficient	$F_{t}$	Tangential friction force
$F_{W}$	Vertical force	$v_{S}$	Slip speed
$v_{p}$	Tire peripheral velocity	v	Velocity
s	Slip	r	Tire radius
ω	Tire angular velocity	$T_{0}$	Engine thrust
$F_{X}$	Aerodynamic drag	$F_{p}$	Drag parachute resistance
$F_{Y}$	Aerodynamic lift	$F_{n}$	Front-wheel load
$F_{z}$	Main wheel load	$F_{f}$	Bonding force between wheel and runway
n	Number of the brake wheels	$J_{w}$	Moment of inertia of the wheel
$B_{w}$	Friction coefficient of an axle	$T_{b}$	Braking torque
$r_{d}/r_{s}$	Radius of the brake disc (rotor/ stator assembly)	$\mu_{f}$	Friction coefficient of the brake disc
$n_{f}$	Number of friction surfaces	$P_{A}$	Pressure applied to the brake disc
$K_{b}$	Torque-pressure conversion coefficient	m	Aircraft weight
$D_{thrust}$	Force caused by thrust	$D_{brakes}$	Force contribution from wheels
$D_{aero}$	Aerodynamic drag	L	Aerodynamic lift
$\mu_{B}$	Effective aircraft braking coefficient	ε	Runway slope
g	Gravitational constant	$R^{2}$	Correlation coefficient
$\mu_{aircraft}$	Aircraft braking coefficient	$\mu_{ground}$	Ground friction coefficient
$\mu_{Brak\_X}^{AC}$	Aircraft braking coefficient under different models		
h	Vertical height between aircraft center of gravity and runway		
$h_{p}$	Vertical distance between drag parachute and aircraft center of gravity		
a	Horizontal distance between aircraft center of gravity and front wheel		
b	Horizontal distance between aircraft center of gravity and rear wheel		
